# The E2F-DP1 Transcription Factor Complex Regulates Centriole Duplication in *Caenorhabditis elegans*

**DOI:** 10.1534/g3.115.025577

**Published:** 2016-01-12

**Authors:** Jacqueline G. Miller, Yan Liu, Christopher W. Williams, Harold E. Smith, Kevin F. O’Connell

**Affiliations:** * Laboratory of Biochemistry and Genetics, National Institute of Diabetes and Digestive and Kidney Diseases, National Institutes of Health, Bethesda, Maryland 20892; † Genomics Core, National Institute of Diabetes and Digestive and Kidney Diseases, National Institutes of Health, Bethesda, Maryland 20892

**Keywords:** centriole duplication, *C. elegans*, transcriptional regulation, E2F/DP1, SAS-6

## Abstract

Centrioles play critical roles in the organization of microtubule-based structures, from the mitotic spindle to cilia and flagella. In order to properly execute their various functions, centrioles are subjected to stringent copy number control. Central to this control mechanism is a precise duplication event that takes place during S phase of the cell cycle and involves the assembly of a single daughter centriole in association with each mother centriole . Recent studies have revealed that posttranslational control of the master regulator Plk4/ZYG-1 kinase and its downstream effector SAS-6 is key to ensuring production of a single daughter centriole. In contrast, relatively little is known about how centriole duplication is regulated at a transcriptional level. Here we show that the transcription factor complex EFL-1-DPL-1 both positively and negatively controls centriole duplication in the *Caenorhabditis elegans* embryo. Specifically, we find that down regulation of EFL-1-DPL-1 can restore centriole duplication in a *zyg-1* hypomorphic mutant and that suppression of the *zyg-1* mutant phenotype is accompanied by an increase in SAS-6 protein levels. Further, we find evidence that EFL-1-DPL-1 promotes the transcription of *zyg-1* and other centriole duplication genes. Our results provide evidence that in a single tissue type, EFL-1-DPL-1 sets the balance between positive and negative regulators of centriole assembly and thus may be part of a homeostatic mechanism that governs centriole assembly.

Centrioles are cylindrical microtubule-based organelles that direct formation of centrosomes and cilia (Winey and O’Toole 2014). Dividing cells possess one or two pairs of centrioles, with each pair containing a newer (daughter) centriole oriented orthogonally to an older (mother) centriole. In this cellular context, centriole pairs are enveloped by a proteinaceous matrix called the pericentriolar material or PCM, thereby forming centrosomes, the cell’s primary microtubule-organizing center (MTOC). Centrosomes mediate intracellular transport, establish cell polarity, and organize the poles of the mitotic spindle to aid in the segregation of chromosomes. In noncycling cells, centrioles shed their PCM and move to the plasma membrane where the mother centriole serves as a basal body to organize cilia and flagella, structures important for cell motility and cell signaling.

Given the crucial roles of centrioles in both cycling and noncycling cells, it is not surprising that aberrations in centriole number or structure have been linked to disease. An excess number of centrioles is found in many different types of tumor cell, where they can disrupt spindle structure and chromosome segregation leading to aneuploidy (Ganem *et al.* 2009). Excess centrioles can also interfere with cilia-based cell signaling (Mahjoub and Stearns 2012) and promote cell migration and invasive behavior (Godinho *et al.* 2015). Thus, excess centrioles can impact the growth of cells in multiple ways. Beyond cancer, defects in centriole structure or number have been linked to several human diseases including autosomal recessive primary microcephaly, primordial dwarfism, and a collection of disorders called ciliopathies (Chavali *et al.* 2014).

In dividing cells, centriole number is maintained through a precise duplication event in which each mother centriole gives rise to one, and only one, daughter centriole during S phase (Firat-Karalar and Stearns 2014). As each centriole pair will form a spindle pole during the ensuing M phase, stringent control of centriole assembly helps ensure spindle bipolarity and the fidelity of cell division. Forward and reverse genetic studies in the nematode *Caenorhabditis elegans* have led to the identification of a set of five core factors that are required for centriole duplication (O’Connell *et al.* 2001; Kirkham *et al.* 2003; Leidel and Gönczy 2003; Kemp *et al.* 2004; Pelletier *et al.* 2004; Delattre *et al.* 2004; Dammermann *et al.* 2004; Leidel *et al.* 2005; Kitagawa *et al.* 2011a; Song *et al.* 2011). Functional orthologs of each of these factors have since been identified in other species including flies and humans, thereby establishing the broad evolutionary conservation of the centriole duplication pathway (Leidel *et al.* 2005; Habedanck *et al.* 2005; Bettencourt-Dias *et al.* 2005; Basto *et al.* 2006; Kleylein-Sohn *et al.* 2007; Rodrigues-Martins *et al.* 2007; Vladar and Stearns 2007; Zhu *et al.* 2008; Kohlmaier *et al.* 2009; Stevens *et al.* 2010; Arquint *et al.* 2012; Vulprecht *et al.* 2012).

Centriole assembly is initiated by the recruitment of Polo-like kinase 4 (Plk4) to the site of centriole assembly (Dzhindzhev *et al.* 2010; Cizmecioglu *et al.* 2010; Hatch *et al.* 2010; Slevin *et al.* 2012; Sonnen *et al.* 2013; Kim *et al.* 2013; Shimanovskaya *et al.* 2014). In vertebrates, this step is executed through a direct physical interaction between Plk4 and its centriole receptors SPD-2 and Cep152. A simpler mechanism operates in worms, where SPD-2 is solely involved in recruiting the Plk4 relative ZYG-1(Delattre *et al.* 2006; Pelletier *et al.* 2006). ZYG-1/Plk4 then recruits the coiled-coil domain containing proteins SAS-6 and SAS-5/Stil. The molecular details of this step appear species-specific but involve a direct physical interaction between Plk4/ZYG-1 and either SAS-5 or SAS-6, and subsequent phosphorylation (Lettman *et al.* 2013; Dzhindzhev *et al.* 2014; Arquint *et al.* 2015; Kratz *et al.* 2015; Moyer *et al.* 2015). At the assembling centriole, SAS-6 dimers oligomerize to form the centriole scaffold, an elegant cartwheel structure in humans and flies or a simpler central tube-like structure in worms (Kitagawa *et al.* 2011b; van Breugel *et al.* 2011). Finally, the coiled-coil containing protein SAS-4 is recruited to the nascent centriole and is required for the assembly of the microtubules of the outer wall (Pelletier *et al.* 2006; Dammermann *et al.* 2008; Schmidt *et al.* 2009).

While many of the molecular details of centriole assembly have been elucidated by recent structural and biochemical studies, many mysteries regarding the regulation of this process remain. In particular, it is not clear how a mother gives birth to a single daughter centriole during each round of duplication. Overexpression/overactivation of the core duplication factors ZYG-1/Plk4 or SAS-6 result in the production of multiple daughter centrioles (Habedanck *et al.* 2005; Peel *et al.* 2007; Kleylein-Sohn *et al.* 2007; Basto *et al.* 2008; Peters *et al.* 2010), indicating that careful regulation of the levels and/or activity of these factors plays a role in limiting the number of daughters assembled during each round of duplication. More recently, a number of studies have shed light on the importance of posttranslational mechanisms in regulating centriole duplication; both the levels of Plk4/ZYG-1 and SAS-6 are stringently controlled by regulated proteolysis (Strnad *et al.* 2007; Cunha-Ferreira *et al.* 2009; Rogers *et al.* 2009; Puklowski *et al.* 2011; Peel *et al.* 2012; Čajánek *et al.* 2015).

Little is known about how centriole duplication is controlled at the level of transcription. In 1999, Meraldi and colleagues showed that the heterodimeric transcription factor E2F-DP played a role in regulating the reduplication of centrioles in S-phase arrested CHO cells (Meraldi *et al.* 1999). However, the relevant genes targeted by E2F were not identified. More recently, several isoforms of the E2F transcription factor family (E2F4 and E2F5), along with their binding partner DP and a cell-specific coregulator multicillin, were found to directly activate the transcription of the core centriole duplication factors in multicilliate cells to upregulate centriole biogenesis (Ma *et al.* 2014). In fact, activation of this transcriptional complex was required for multicilliate cell differentiation. In contrast to the positive role for E2F4 and E2F5 in multicilliate cells, a negative role for E2F3 was demonstrated in mouse embryonic fibroblasts (MEFs). Specifically, inactivation of E2F3, but not other isoforms of E2F, in MEFs resulted in centrosome amplification (Saavedra *et al.* 2003). These studies show that E2F-DP may play either a positive or negative role in regulating centriole duplication, with the nature of the role appearing to depend upon the cell type and specific isoform of E2F. Here, we show that E2F-DP also plays a role in regulating centriole duplication in *C. elegans* embryos. Remarkably, we find that E2F-DP plays both a positive and a negative role in a single cell type and propose that E2F-DP1 controls the balance of positive and negative regulators of centriole assembly.

## Materials and Methods

### Worm maintenance and strains

Worm strains were cultivated using standard practices (Brenner 1974) at 20° on MYOB plates seeded with OP50 *Escherichia coli*. A complete list of the strains used in this work can be found in Supporting Information, Table S2. Suppression of the *zyg-1(it25)* phenotype was assayed at 24° or 23.5°, as indicated. Scatter plots displaying suppression data were generated using the Excel templates provided by Weissgerber *et al.* (2015).

Transgenic worm strains were made using Mos1-mediated single copy insertion (MosSCI) transformation (Frøkjaer-Jensen *et al.* 2008). pKO113, the *zyg-1* transcriptional reporter construct, was generated using Gateway cloning technology (Thermo Fisher Scientific, Inc., Waltham MA) and contained the wild-type *zyg-1* promoter (1082 nucleotides upstream of the transcriptional start site), a *gfp-his-58* fusion gene, and the *zyg-1* 3′ UTR cloned into the MosSCI targeting vector pCFJ210 (Frøkjær-Jensen 2012). An identical approach was used to construct pKO114, except that the Quikchange II site-directed mutagenesis kit (Agilent Technologies, Inc., Santa Clara, CA) was used to mutate the three EFL-1-DPL-1 binding sites in the *zyg-1* promoter entry clone prior to Gateway cloning.

### Mutation identification

Molecular identification of suppressor mutations was accomplished by combining different mapping strategies with whole-genome sequencing. The preparation of genomic DNA, construction of sequencing libraries, and generation of sequence data were essentially as described previously (Wang *et al.* 2014). Variants were identified using a pipeline of BFAST (Homer *et al.* 2009), SAMTools (Li *et al.* 2009), and ANNOVAR (Wang *et al.* 2010). Mapping plots were generated using R (R Core Development Team, 2015). Candidate suppressor alleles were limited to homozygous (minimum three independent reads, ≥ 85% variant call), nonsynonymous mutations, and filtered to remove variants common to the strain background.

For *dpl-1(bs21)*, the suppressor mutation was mapped by classical three-factor mapping between *dpy-10* and *unc-4* on linkage group II (Kemp *et al.* 2007). The strain containing *bs21* (OC204) was sequenced, and candidate suppressors in the *dpy-10* -*unc-4* interval were identified. For *mat-3(bs29)*, the original map position on linkage group II (Kemp *et al.* 2007) was found to be incorrect (data not shown). The position of this suppressor was reinvestigated, and three-factor mapping with *unc-45* and *dpy-1* revealed that *bs29* was tightly linked to *unc-45* on the left arm of chromosome III. The strain containing *bs29* (OC184) was sequenced to identify candidate suppressors in the vicinity of *unc-45*. For *efl-1(bs22)*. A variation of the one-step method for simultaneous mapping and sequencing was employed (Doitsidou *et al.* 2010). The *bs22* suppressor mutation was introgressed into the Hawaiian CB4856 background by backcrossing 10 successive times to a Hawaiian-introgressed *zyg-1(it25)* strain. Mapping plots of Hawaiian SNPs across the genome revealed (in addition to the interval flanking *zyg-1* on chromosome II) gaps on chromosome I (between 2.0–3.0 Mb) and chromosome V (from 15.0 Mb to the right end). The chromosome I interval encompasses a known locus of genetic incompatibility (*zeel-1/peel-1* at 2.35 Mb) between the N2 wild-type and Hawaiian strain backgrounds (Seidel *et al.* 2008) and was not pursued further. Candidate suppressors were identified in the chromosome V interval.

### Genome editing

For genome editing, we utilized coCRISPR technology essentially as described (Arribere *et al.* 2014). Specifically, we designed guide RNAs (gRNAs) using the CRISPR Design tool at http://crispr.mit.edu. gRNA sequences were inserted into the expression plasmid pDD162 (Dickinson 2013) using the Q5 Site Directed Mutagenesis Kit (New England Biolabs, Inc., Ipswich, MA). All oligos used can be found in Table S3. All constructs were sequence verified. For repair templates, we used single stranded oligomers (Paix *et al.* 2014) synthesized by Integrated DNA Technologies, Inc. (Coralville, IA). For microinjection, we prepared a mixture of two *efl-1* gRNA expression plasmids at 50 ng/μl each, purified *efl-1* repair template at 30 ng/μl, the *dpy-10* coconversion gRNA expression plasmid at 50 ng/μl, and the *dpy-10* repair template at 20 ng/μl.

After injection, P_0_ hermaphrodites were transferred to individual MYOB plates at 20° and allowed to produce an F1 generation. F1 progeny exhibiting a Rol or Dpy phenotype were picked individually to MYOB plates and allowed to lay F3 eggs. F2 adults were then picked individually to a PCR tube, lysed, then screened by PCR for the loss (wt > mut) or gain (mut > wt) of an *Nla*IV restriction site affected by the *bs22* mutation (underlined residues in the *efl-1* gRNA sequences in Table S3).

### RNAi

RNAi experiments were carried out by feeding worms *E. coli*-expressing inducible dsRNA constructs as previously described (Kamath *et al.* 2003). L4 larvae were seeded onto fresh RNAi plates and the effects of RNAi were monitored 12–24 hr from the L4 stage. The L4440 vector (Source Bioscience, Nottingham, UK), expressing dsRNA against the *smd-1* gene, was used as a negative control for all RNAi experiments.

### Antibodies and quantitative immunoblotting

Embryos were isolated by washing worms off four 10 cm plates and suspending them in a hypochlorite solution (1.65% hypochlorite, 1 M NaOH) for ∼5 min. Once adult worms were dissolved, embryos were rinsed 3 times using M9 buffer, suspended in ∼50 μl 2 x LDS Sample buffer (Life Technologies, Inc., Carlsbad, CA) and heated to 95° for 5 min. Samples were resolved on NuPage Bis-Tris gels (Life Technologies, Inc., Carlsbad, CA) and transferred to nitrocellulose using the i-Blot transfer system (Thermo Fisher Scientific, Waltham, MA). Blots were probed and analyzed using the Odyssey Infrared Imaging System (LI-COR Biosciences, Inc., Lincoln, NE) as previously described (Song *et al.* 2011).

The following antibodies were used at a dilution of 1:500 – 1:2000: DM1A, an α-tubulin specific antibody (Sigma), α-SPD-2 (Kemp *et al.* 2004), α-ZYG-1 (Kemp *et al.* 2007), and α-SAS-4 (Song *et al.* 2008). The SAS-6 antibody is a polyclonal antibody raised in guinea pigs to a full-length Glutathione-S-Transferase-SAS-6 fusion protein. The antibody was produced by Pocono Rabbit Farm and Laboratory, Inc. (Canadensis, PA) and affinity purified against a full-length Maltose-Binding Protein-SAS-6 fusion protein. Antibody to a SAS-5-derived peptide (N-CPAERERRIREKYARRK-C) was raised in rabbits and affinity purified by YenZym Antibodies LLC, (San Francisco, CA). IRDye secondary antibodies (LI-COR Biosciences) were used at 1:15,000 and membranes were imaged using the Odyssey Infrared Imaging System (LI-COR Biosciences). Bands were normalized to an α-tubulin loading control and quantitated using Image-J software (NIH, Bethesda, MD). Graphs depict the average normalized protein levels from 3 independent experiments.

### qRT-PCR

To isolate RNA, L4 worms were transferred to 25° overnight. Approximately 50–100 adult worms were suspended in 200 μl M9 buffer, and RNA was isolated using the Arcturus PicoPure RNA Isolation Kit (Thermo Fisher Scientific, Inc., Waltham, MA) according to the manufacturer’s instructions. RNA was treated with DNAase and then diluted to 20 ng/μl using RNAase-free ddH_2_O. qRT-PCR reactions were set up in triplicate using 20 ng of template RNA, a QuantiFast SYBR Green RT-PCR Kit from Qiagen (Valencia, CA), and 10 μM primers (see Table S4 for primer sequences). A negative control lacking reverse transcriptase was set up for each RNA template and a nontemplate control was set up for each primer set. Quantitative RT-PCR was performed on a CFX96 Touch Real-Time PCR Detection System (Bio-Rad Laboratories, Inc., Hercules, CA). Amplification reactions were carried out using the following program: 10 min at 50°, 5 min at 95° and then 40 cycles (10 sec at 95° and 30 sec at 55°). A melting curve was determined at the end of each PCR run to verify the formation of a single amplicon. Average Ct values were determined using CFX Manager 3.0 Software and the fold change was calculated using the 2^ΔΔCt^ method. For each primer set, the Ct values were normalized to *tba-1* RNA levels and then compared to a wild-type calibrator sample. The error bars indicate the SEM of the triplicate set.

### Microscopy and live cell imaging

Centriole duplication was monitored using 4D time-lapse microscopy on a Nikon spinning disc confocal microscope as previously described (Peters *et al.* 2010). Images for comparing the expression of the *zyg-1* transcriptional reporters were taken on the same day using the same camera settings.

### Data availability

Sequence data are available from the Sequence Read Archive (SRA) under BioProject PRJNA309986.

## Results

### Mutation of szy-10 suppresses embryonic lethality and restores centriole duplication in the zyg-1(it25) mutant

**Figure 1:**
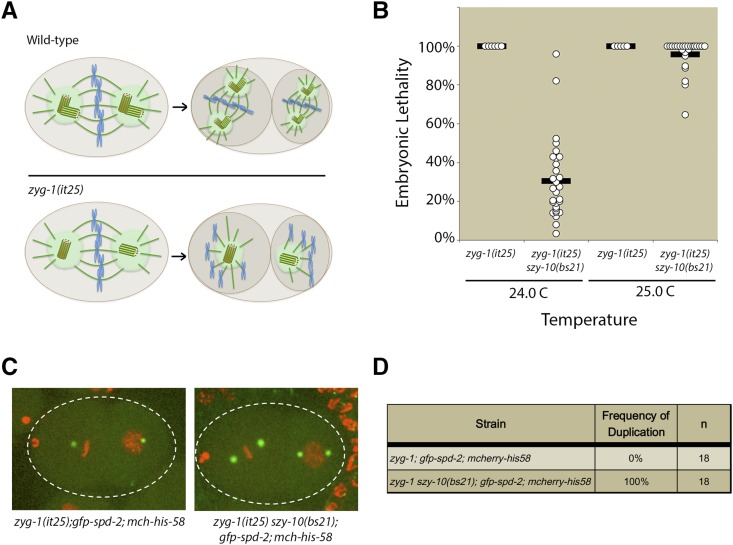
*szy-10(bs21)* suppresses embryonic lethality and centriole duplication defects of the *zyg-1(it25)* mutant. (A) In wild-type embryos, sperm-derived centrioles duplicate during the first cell cycle resulting in the formation of bipolar spindles at the two-cell stage. In *zyg-1(it25)* mutants, the sperm-derived centrioles fail to duplicate, leading to monopolar spindles at the two-cell stage. (B) *zyg-1(it25)* or *zyg-1(it25) szy-10(bs21)* mutants were shifted to the nonpermissive temperature (24° or 25°) at the L4 larval stage and embryonic lethality of their self-progeny was quantified. (C) *zyg-1(it25)*; *mcherry-his-58*; *gfp-spd-2* or *zyg-1(it25) szy-10(bs21)*; *mcherry-his-58*; *gfp-spd-2* animals were shifted to the nonpermissive temperature of 24° at the L4 larval stage and allowed to grow for 24 hr. Centriole duplication in their embryos was monitored using time-lapse microscopy. Representative images of control *zyg-1(it25)* or *zyg-1(it25) szy-10(bs21)* embryos at the two-cell stage are shown. (D) Quantification of centriole duplication in *zyg-1(it25)*; *mcherry-his-58*; *gfp-spd-2* or *zyg-1(it25) szy-10(bs21)*; *mcherry-his-58*; *gfp-spd-2* reporter strains. n=number of events.

The *szy-10* gene was initially identified as a genetic suppressor of the temperature-sensitive *zyg-1(it25)* mutant (Kemp *et al.* 2007). At the nonpermissive temperature of 24°, embryonic centriole duplication fails in the *zyg-1(it25)* mutant. As a result, the pair of centrioles derived from the sperm separate and establish a bipolar mitotic spindle during the first embryonic division. The absence of centriole duplication during the first cell cycle results in each daughter cell inheriting only a single centriole, which goes on to organize a monopolar spindle in each blastomere of the two-cell embryo (Figure 1A). This failure in centriole duplication also results in a fully penetrant embryonic lethal phenotype at 24°. In contrast to the *zyg-1(it25)* single mutant, a *zyg-1(it25) szy-10 (bs21)* double mutant was able to produce a significant number of viable progeny at 24° (Figure 1B). At the slightly higher temperature of 25°, very little suppression was observed in the *zyg-1(it25) szy-10(bs21)* double mutant (Figure 1B). As the *zyg-1(it25)* mutant is significantly more impaired at 25°, the failure of suppression at the higher temperature indicates that the *szy-10(bs21)* mutation does not bypass the requirement for *zyg-1* in centriole duplication. Rather, the *szy-10(bs21)* mutation likely either elevates the residual ZYG-1 activity in the mutant, or alternatively, eases the requirement for ZYG-1 by facilitating execution of the pathway downstream of ZYG-1.

To investigate whether *szy-10* suppresses *zyg-1* embryonic lethality by restoring centriole duplication, time-lapse microscopy was performed in *zyg-1(it25)* and *zyg-1(it25) szy-10(bs21)* mutants expressing GFP-labeled SPD-2 as a marker for the centrosomes and mCherry-labeled histone H2B as a proxy for the DNA. As expected, centriole duplication invariably failed in *zyg-1(it25)* mutants leading to monopolar spindles at the two-cell stage (Figure 1, C and D). However, in the *zyg-1(it25) szy-10(bs21)* double mutant, all centrosomes successfully duplicated to generate bipolar spindles at the two-cell stage (Figure 1, C and D). These results demonstrate that *szy-10(bs21)* suppresses embryonic lethality by restoring centriole duplication in the *zyg-1(it25)* mutant.

To determine if mutation of maternal *szy-10* is sufficient for suppression of the *zyg-1(it25)* centriole duplication defect or whether there is a paternal contribution, *zyg-1(it25) szy-10(bs21)* hermaphrodites were mated to *zyg-1(it25)* males and centriole duplication was analyzed in the resulting progeny. The centrosomes duplicated in three out of three embryos, indicating that perturbation of maternal SZY-10 is sufficient for suppression of *zyg-1(it25)* (data not shown). Thus, paternal expression of the mutant *szy-10* gene does not appear to contribute to suppression of the *zyg-1(it25)* centriole duplication defect.

**Figure 2:**
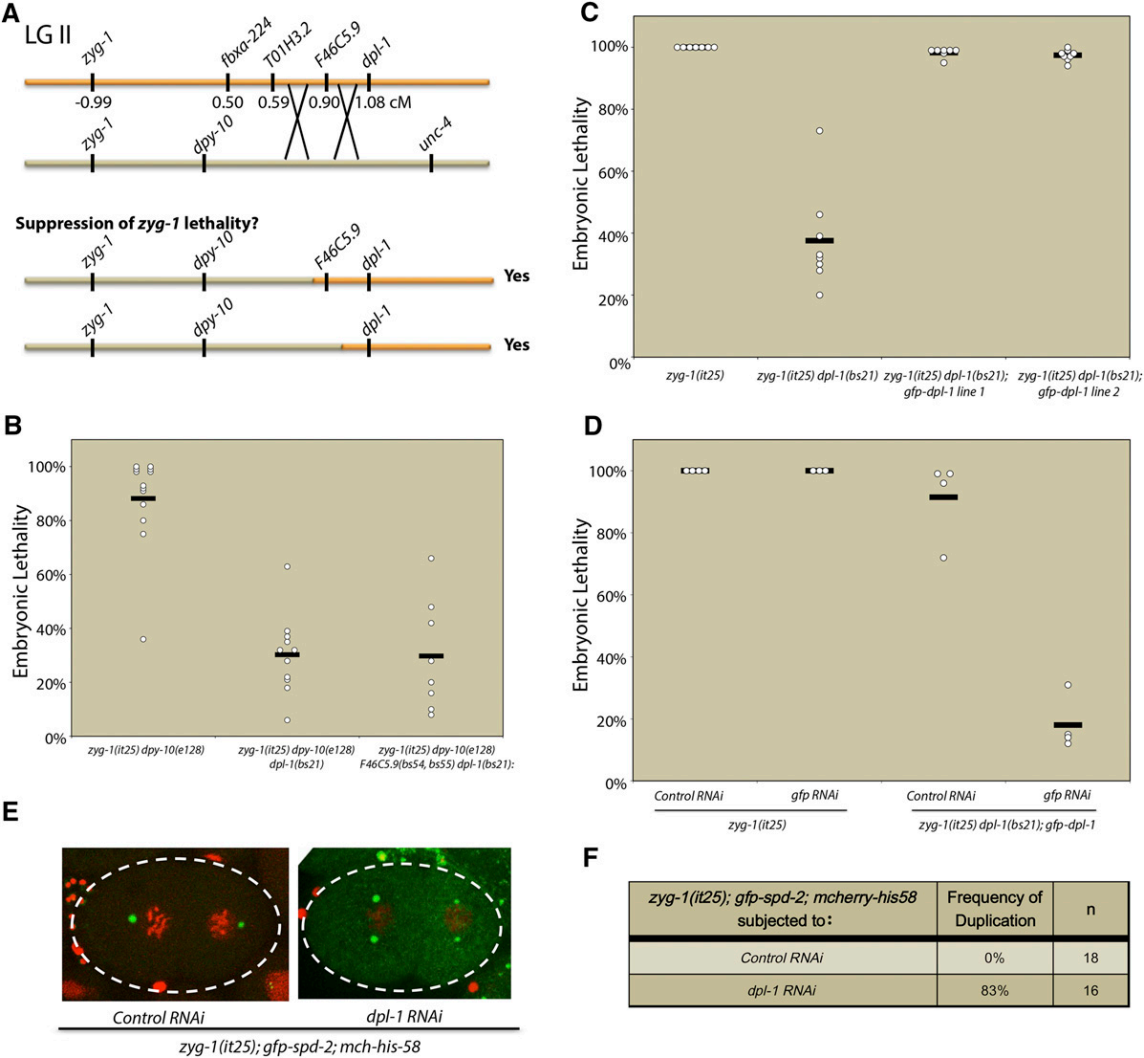
The *bs21* mutation is an allele of the *dpl-1* gene. (A) Schematic of recombination mapping of the *szy-10(bs21)* mutation. (B) Embryonic lethality of the *zyg-1(it25) dpy-10(e128)* or recombinants containing mutations in one or more of the *szy-10* candidate genes was quantified at the nonpermissive temperature of 24°. (C) A *gfp-dpl-1* transgene complements *dpl-1(bs21)*-mediated suppression of the *zyg-1(it25)* embryonic lethal phenotype. The graph plots the embryonic lethality at 24° of *zyg-1(it25)* control animals and *zyg-1(it25) dpl-1(bs21)* animals with and without a *dpl-1-gfp* transgene. (D) *zyg-1(it25) dpl-1(bs21)*; *dpl-1-gfp* animals were treated with control or *gfp* RNAi and embryonic lethality was tested at 24°. (E) *zyg-1(it25)*; *mcherry-his58*; *gfp-spd-2* L4 animals were treated with control RNAi or dpl-1 RNAi for 24 hr and the frequency of centriole duplication was monitored by time-lapse microscopy. Representative images of two-cell embryos treated with control RNAi (left) or *dpl-1* RNAi (right) are shown. (F) Quantification of centriole duplication events in *zyg-1(it25)*; *mcherry-his-58*; *gfp-spd-2* animals treated with control or *dpl-1* RNAi.

To determine the molecular identity of the *szy-10* gene, a combination of traditional three-factor mapping and whole-genome sequencing was employed. The *szy-10* gene had previously been mapped to a region between *dpy-10* and *unc-4* on chromosome II (Kemp *et al.* 2007). Whole-genome sequencing of the *zyg-1(it25) szy-10(bs21)* double mutant identified just four mutations affecting splice-sites or effecting nonsynonymous amino acid changes within this interval (Figure 2A and Figure S1A). Higher resolution mapping definitively demonstrated that the mutation responsible for suppression of the *zyg-1(it25)* phenotype was a mutation in the *dpl-1* gene. Specifically, *zyg-1(it25) dpy-10(e128) unc-4(e120)/zyg-1(it25) + szy-10(bs21) +* hermaphrodites were constructed, and F1 Dpy nonUnc and Unc nonDpy recombinants were isolated (Figure 2A and Figure S1B). Recombinants containing the mutant *dpl-1* allele exhibited suppression of the *zyg-1(it25)* embryonic lethality (Figure 2B), while the presence of the other mutations did not correlate with suppression. Conversely, recombinants that had lost the *dpl-1* mutation but retained the other three mutated genes failed to suppress *zyg-1(it25)* (Figure S1B). Taken together, these results indicate that mutation of *dpl-1* is necessary and sufficient for suppression of the *zyg-1* phenotype. To further confirm the identity of the gene, we used molecular complementation; specifically, we found that introduction of a *dpl-1-gfp* transgene into the *zyg-1(it25) szy-10(bs21)* strain resulted in loss of suppression (Figure 2C). RNAi-mediated depletion of the transgene using RNAi directed against *gfp* reversed these effects, ensuring that the loss of suppression was due to the presence of the extra copies of *dpl-1* rather than any other genetic variances introduced by the transgenic strain (Figure 2D). We conclude that the *bs21* mutation is an allele of the *dpl-1* gene and hereafter refer to this gene as *dpl-1*.

The complementation of *dpl-1(bs21)* by a wild-type *dpl-1* transgene indicated that *dpl-1(bs21)* is a loss-of-function mutation. Consistent with this finding, RNAi-mediated depletion of *dpl-1* in the *zyg-1(it25)*; *gfp-spd-2*; *mCherry-h2b* strain restored centriole duplication (Figure 2, E and F). While centriole duplication never occurred in *zyg-1(it25)* animals treated with control RNAi, centrosome duplication occurred ∼80% of the time in *zyg-1(it25)* animals treated with *dpl-1* RNAi (Figure 2F). These results confirmed that a loss of DPL-1 function mediated either by mutation or RNAi suppresses the *zyg-1(it25)* centriole duplication defect and embryonic lethality, and indicates that DPL-1 is a negative regulator of centriole duplication.

### Inhibition of the C. elegans E2F-DP1 transcription factor suppress zyg-1(it25) defects

The *dpl*-1 (DP-like) gene encodes a conserved transcription factor that is required for the G1-to-S-phase cell cycle transition in higher eukaryotes. DP heterodimerizes with a member of the E2F family of transcription factors and, in some cases, the E2F-DP1 heterodimer interacts with the tumor suppressor protein Retinoblastoma (Rb) to regulate a number of genes involved in the G1-S cell cycle transition. In *C. elegans*, this family of transcriptional regulators also controls cell cycle progression (Park and Krause 1999; Boxem and van den Heuvel 2001, 2002: Fay *et al.* 2002); however, a more predominant role has been elucidated in the control of developmentally-regulated processes such as vulval development, oocyte maturation, and early embryogenesis (Ceol and Horvitz 2001; Page *et al.* 2001; Chi and Reinke 2006; Kirienko and Fay 2007).

**Figure 3:**
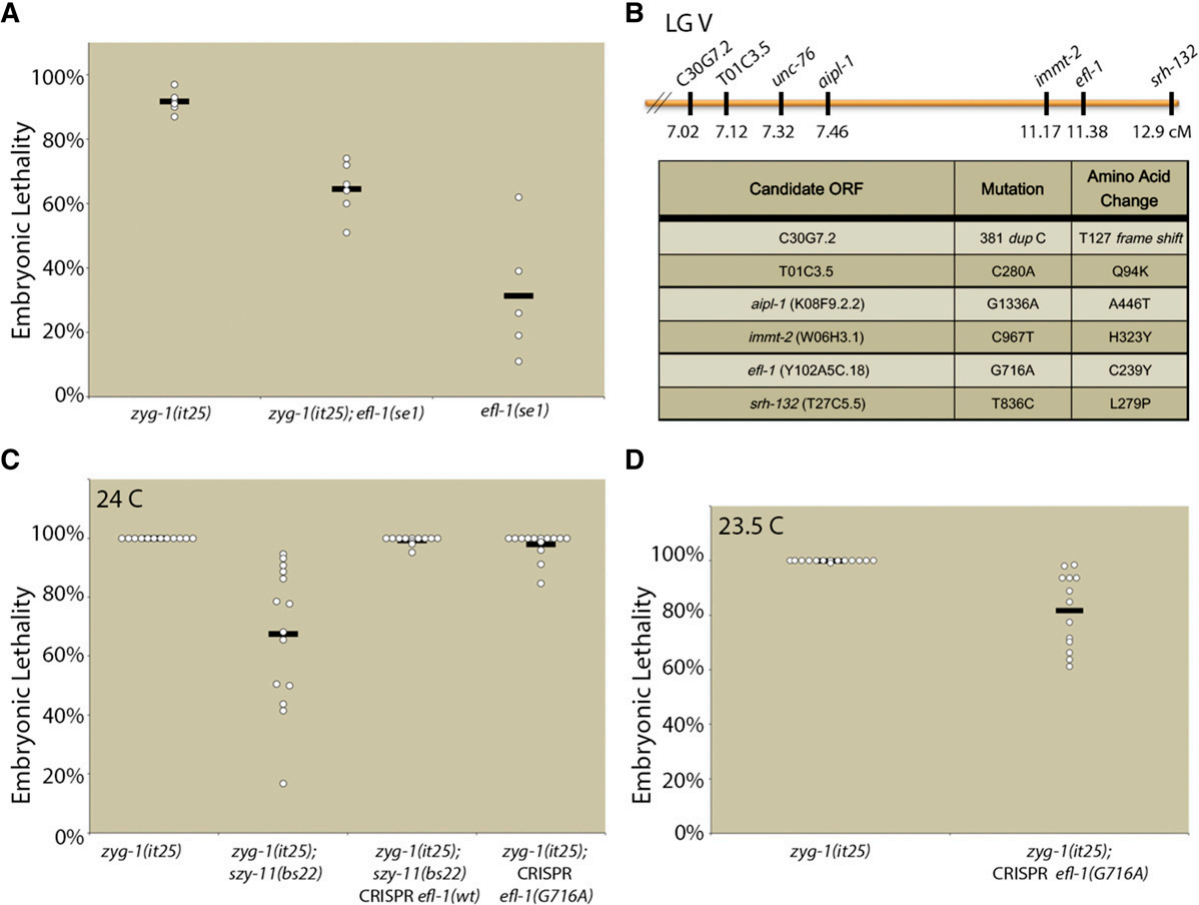
Mutation of *efl-1* suppresses *zyg-1(it25)* embryonic lethality. (A) *zyg-1(it25)*, *zyg-1(it25); efl-1(se1)*, and *efl-1(se1)* were shifted to 25° as gravid adults. Shown are the levels of embryonic lethality for the ensuing 24 hr. (B) *szy-11(bs22)* was mapped in the vicinity of *unc-76* on the right arm of LG V. Shown are the identities and map positions of linked mutations resulting in nonsynonymous codon changes. (C) *szy-11(bs22)*-mediated suppression of *zyg-1(it25)* requires the *efl-1* mutation. The *efl-1* mutation was reverted to the wild-type sequence in the *zyg-1(it25)*; *szy-11(bs22)* strain using CRISPR technology and suppression of *zyg-1(it25)* embryonic lethality was quantitated. Additionally, the *efl-1* mutation (*G716A*) was introduced into the *zyg-1(it25)* mutant and suppression of embryonic lethality was measured at 24°. (D) Quantitation of embryonic lethality of *zyg-1(it25) or zyg-1(it25)*; CRISPR *efl-1(G716A)* at 23.5°.

To determine whether the suppression of *zyg-1(it25)* centriole duplication defects was due to effects on the transcription factor activities of DPL-1, we tested whether loss of its heterodimerization partner EFL-1 would also suppress the *zyg-1(it25)* phenotype. Because null mutants of *efl-1* are sterile, we utilized the conditional partial loss-of-function allele *efl-1(se1)* to test for genetic interaction between these factors. The *efl-1(se1)* mutant exhibits two temperature-sensitive periods. Shifting the mutant to the nonpermissive temperature of 26° prior to the L4 stage results in sterility, while shifting after the L4 stage results in maternal-effect embryonic lethality. In order to determine whether loss of *efl-1* function could suppress *zyg-1(it25)*, we shifted gravid adults to the nonpermissive temperature of 25° and allowed them to lay eggs for 24 hr. The adults were then removed and the embryos allowed to develop for 24 hr. Under these experimental conditions, *zyg-1(it25)* mutants exhibited an average of 92% embryonic lethality, while *efl-1(se1)* mutants exhibited an average of 31% embryonic lethality (Figure 3A). The *zyg-1(it25)*; *efl-1(se1)* double mutants exhibited an average of 64% embryonic lethality. This result indicates a strong positive epistatic interaction, as for two noninteracting mutations the fitness of the double mutant should simply be the product of the fitness values of each single mutant (Beltrao *et al.* 2010). Thus, if *zyg-1(it25)* and *efl-1(se1)* were noninteracting mutations, we would expect the double mutant to exhibit a fitness of 0.06 (94% embryonic lethality): 0.08 [fitness of *zyg-1(it25)*] × 0.69 [fitness of *efl-1(se1)*] = 0.06. However, the fitness of the double mutant was 0.36 (36% viability with a standard deviation of 8.4%), more than three standard deviations above the expected value. We conclude that a loss of either member of the EFL-1-DPL-1 transcription factor complex suppresses *zyg-1(it25)* defects, implicating either a direct or indirect role for transcriptional regulation by EFL-1-DPL-1 in the control of centriole duplication.

Further corroborating a role for the EFL-1-DPL-1 transcriptional complex in regulating centriole duplication, we independently identified an allele of *efl-1* as a genetic suppressor of *zyg-1(it25)*. The *szy-11* gene had previously been mapped to the vicinity of *unc-76* on linkage group V (LG V, Kemp *et al.* 2007). We outcrossed the *zyg-1(it25)*; *szy-11(bs22)* line to a Hawaiian polymorphic line carrying the *zyg-1(it25)* mutation 10 successive times. Whole-genome sequencing of this Hawaiian-ingressed *zyg-1(it25)*; *szy-11(bs22)* double mutant identified a region on the right arm of LG V that lacked Hawaiian SNPs. Within this region, six open reading frames contained protein-coding mutations (Figure 3B). One of these mutations was located in the *efl-1* ORF and is predicted to result in a nonsynonymous codon change. To confirm the molecular identity of the *szy-11(bs22)* suppressor, we used CRISPR technology to revert the mutation in *efl-1* to the wild-type sequence and examined the suppression of *zyg-1(it25)* embryonic lethality. At 24°, *zyg-1(it25)*; *szy-11(bs22)* hermaphrodites are able to produce a variable number of viable offspring (Figure 3C). However, reversion of the *efl-1* mutation in this strain resulted in an essentially complete loss of suppression at the nonpermissive temperature, confirming that *bs22* is an allele of *efl-1*. Curiously, introduction of the *efl-1(bs22)* mutation into the original *zyg-1(it25)* strain provided only very weak suppression at 24° (Figure 3C). However, suppression of *zyg-1(it25)* embryonic lethality by the CRISPR-engineered *efl-1(bs22)* mutation was evident at the less restrictive temperature of 23.5° (Figure 3D). Thus, while the *efl-1(bs22)* mutation provides moderate suppression of *zyg-1(it25)*, evidently other genetic elements present in the original *zyg-1(it25)*; *szy-11(bs22)* strain also contribute to suppression. Nonetheless, these results indicate that both DPL-1 and its heterodimeric partner EFL-1 negatively regulate centriole duplication.

### EFL-1-DPL-1 directly regulates the expression of centriole duplication factors

**Figure 4:**
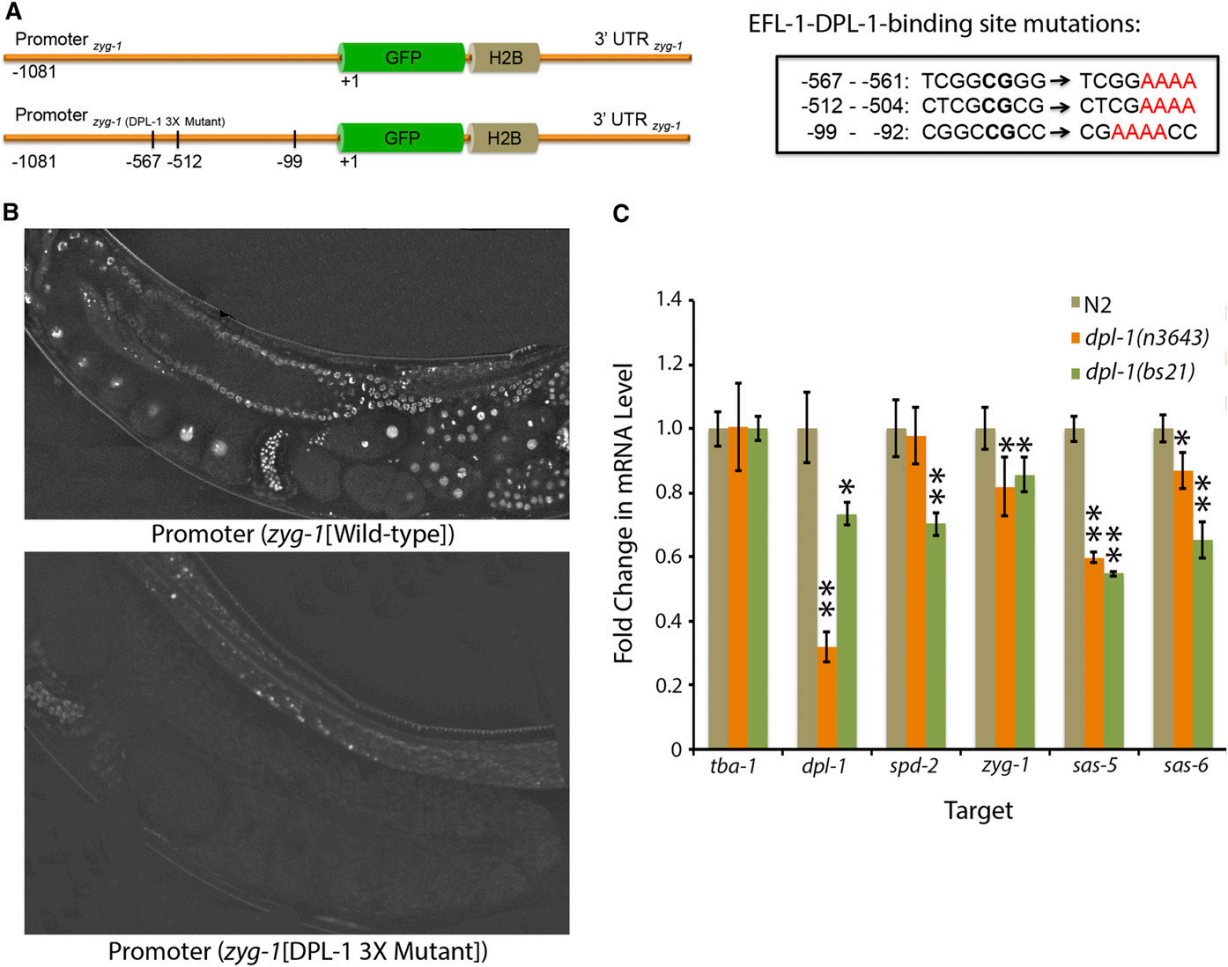
Regulation of transcription of centriole duplication factors by EFL-1-DPL-1. (A) Schematic of the wild-type *zyg-1* transcriptional reporter (top) and the corresponding version with the three EFL-1-DPL-1-binding sites mutated (bottom). Mutations in the EFL-1-DPL-1-binding sites are pictured in the box at right. (B) Representative images of the germ lines of transgenic animals expressing the wild-type *zyg-1* transcriptional reporter (top) or animals expressing the triple mutant binding site reporter (bottom). (C) Relative levels of centriole duplication transcripts in wild-type and *dpl-1* mutant animals. RNA was isolated from wild-type, *dpl-1(n3643)*, or *dpl-1(bs21)* adults, and quantitative real-time PCR was used to analyze RNA levels. Values significantly different from controls were determined using a Student’s *t*-test and are indicated with one (*P* < 0.05) or two (*P* < 0.01) asterisks. H2B, histone H2B; GFP, green fluorescent protein; UTR, untranslated region.

To determine the molecular mechanism by which loss of the EFL-1-DPL-1 transcriptional complex suppresses lethality in the *zyg-1(it25)* strain, we investigated whether this complex directly modulates the levels of *zyg-1* and/or other core duplication factors. Most of the genes encoding core duplication factors, such as *spd-2*, *zyg-1*, *sas-5*, and *sas-6*, contain consensus EFL-1-DPL-1 binding sites within their promoters (Table S1). Moreover, all of these promoters are known to be occupied by both EFL-1 and DPL-1 *in vivo* (Kudron *et al.* 2013). To determine whether EFL-1-DPL-1 directly regulates the expression of *zyg-1*, we generated a transcriptional reporter strain in which the *zyg-1* promoter drives expression of a GFP-labeled histone H2B. The *zyg-1* 3′ UTR was used to direct the translation of this reporter strain (Figure 4A). We also generated a construct containing mutations in all three of the putative EFL-1-DPL-1 binding sites within the *zyg-1* promoter (Figure 4A). Single-copy insertion of the reporter constructs was achieved using the *MosI*-mediated single copy insertion (MosSCI) method of transgenesis (Frøkjær-Jensen *et al.* 2008), and allowed for direct comparison of *zyg-1* expression in the wild-type and EFL-1-DPL-1-binding mutant. Examination of several independent strains expressing the wild-type *zyg-1* reporter revealed that *zyg-1* is produced throughout the germ line and within early embryos. Expression was detected as early as the distal gonad (Figure 4B). If EFL-1-DPL-1 is a negative regulator of *zyg-1* expression, we would expect that a loss of the EFL-1-DPL-1-binding sites within the promoter would result in an increase in *zyg-1* expression. However, we found that mutation of these EFL-1-DPL-1 binding sites resulted in a complete loss of expression of the *zyg-1* reporter (Figure 4B). This result implicates a positive role for EFL-1-DPL-1 in the regulation of *zyg-1* expression within the germ line and early embryo. Thus, our result is consistent with microarray experiments showing that EFL-1-DPL-1 primarily promotes the expression of genes within the germ line (Chi and Reinke 2006).

To further investigate the role for EFL-1-DPL-1 in regulating the expression of centriole duplication genes, we used quantitative real-time PCR to measure transcript levels in the wild-type and *dpl-1* mutants. Specifically, we examined message levels of the core duplication factors (*spd-2*, *zyg-1*, *sas-5*, and *sas-6*) in wild-type animals or in animals carrying either of two partial loss-of-function *dpl-1* mutations: [*dpl-1(bs21)* and *dpl-1(n3643)*]. Consistent with our findings using the *zyg-1* reporter constructs, we found that partial loss of *dpl-1* function resulted in slight to moderate decreases in the message levels of the endogenous centriole duplication genes (Figure 4C). Taken together, these results indicate that EFL-1-DPL-1 likely promotes the expression of several core centriole duplication factors. Therefore, suppression by loss-of-function mutations in *dpl-1* and *efl-1* is not due to an increase in the expression of core duplication factors, but instead is likely mediated indirectly through changes in the expression of an as-of-yet unidentified factor(s).

### Loss of EFL-1-DPL-1 suppresses zyg-1 through an indirect mechanism

**Figure 5:**
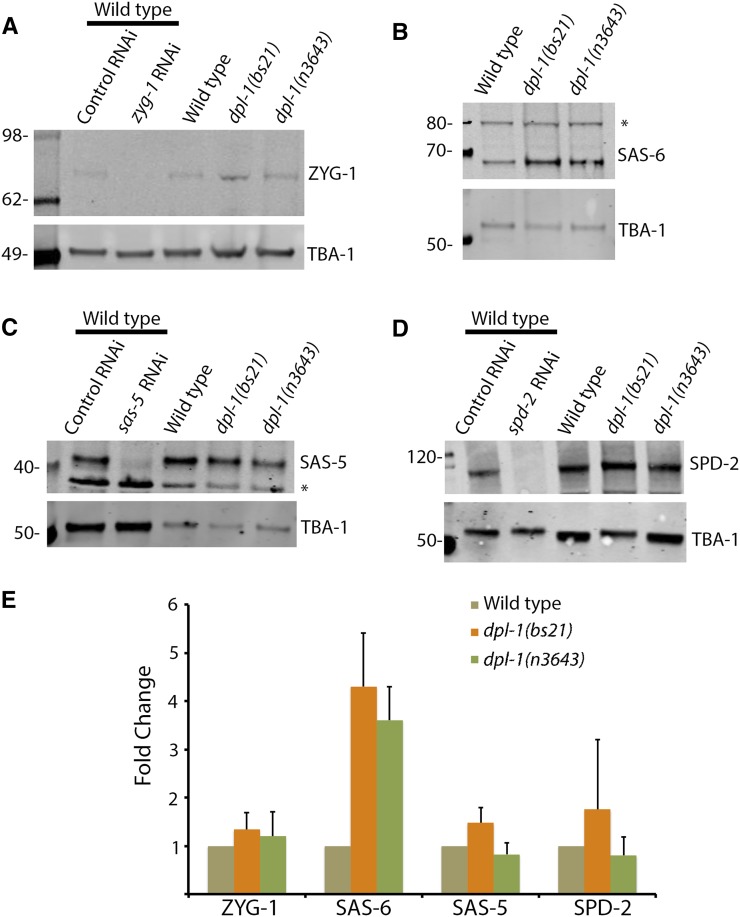
SAS-6 levels are increased in *dpl-1* mutants. (A–D) Immunoblot analysis of extracts made from wild-type, *dpl-1(bs21)*, or *dpl-1(n3643)* embryos. Shown are representative blots probed for (A) ZYG-1, (B) SAS-6, (C) SAS-5, and (D) SPD-2. In each case, α-tubulin was used as a loading control. The specificities of the ZYG-1, SAS-5, and SPD-2 antibodies are demonstrated by the absence of a band in extracts depleted of that specific factor by RNAi. The specificity of the SAS-6 antibody is shown in Figure 6. Asterisks denote nonspecific bands. (E) Relative levels of each factor in the wild-type and the two *dpl-1* mutants. Signals were normalized to the loading control and plotted relative to the wild-type. Each measurement is from two or more independent experiments.

Although loss of EFL-1-DPL-1 activity did not result in an increased level of any of the transcripts encoding centriole duplication factors, we decided to examine the steady-state protein levels of these same factors in animals compromised for EFL-1-DPL-1 function. Specifically, embryonic extracts were prepared from wild-type, *dpl-1(bs21)*, or *dpl-1(n3643)* strains and quantitative immunoblot analysis was performed using antibodies specific for ZYG-1, SPD-2, SAS-5, or SAS-6. Samples were normalized against tubulin, which was used as a loading control. Surprisingly, while *sas-6* message levels were not increased in *dpl-1* mutants, the level of SAS-6 protein was consistently elevated three- to fourfold (Figure 5, A and B). In contrast, no significant changes in the protein levels of ZYG-1, SPD-2, or SAS-5 were detected (Figure 5, A and B). SAS-6 is normally recruited to nascent centrioles by ZYG-1 during the early events of centriole duplication (Delattre *et al.* 2006; Pelletier *et al.* 2006; Lettman *et al.* 2013). It is possible that SAS-6 recruitment is less efficient in the *zyg-1(it25)* mutant, and that overexpression of SAS-6 ameliorates this defect. Thus, the elevated level of SAS-6 in the *dpl-1* mutants provides a possible mechanism by which the loss of *dpl-1* compensates for crippled *zyg-1* activity.

Taken together, our data suggest that EFL-1-DPL-1 regulates centriole duplication in part by downregulating the level of SAS-6 protein. As our results indicate that EFL-1-DPL-1 promotes transcription of *sas-6* and other components of the duplication pathway, the elevated level of SAS-6 protein in the *dpl-1* mutant is likely the indirect effect of altered expression of some yet-to-be-identified factor(s). In vertebrates, SAS-6 levels are regulated by the anaphase promoting complex/cyclosome (APC/C), an E3 ubiquitin ligase that targets various cell cycle proteins for destruction by the proteasome (Strnad *et al.* 2007). Interestingly, Kudron *et al.* (2013) identified several APC/C components as potential targets of EFL-1-DPL-1. Thus, one possible model to explain our results is that the EFL-1-DPL-1 transcriptional complex normally promotes expression of one or more APC/C components that in turn leads to downregulation of SAS-6.

**Figure 6:**
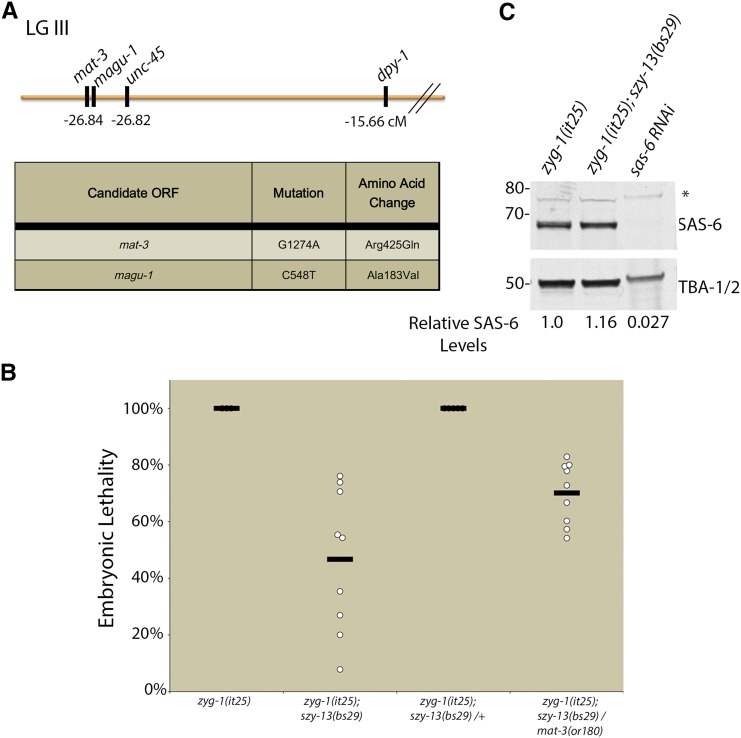
Loss of APC/C activity suppresses *zyg-1(it25)*. (A) *szy-13(bs29)* was mapped relative to *unc-45* and *dpy-1* on the left arm of LG III (map at top). Whole-genome sequencing of this strain revealed just two genes in the vicinity with nonsynonymous codon changes (table at bottom). (B) The molecular identity of *szy-13(bs29)* was confirmed through complementation analysis. Suppression of *zyg-1(it25)* embryonic lethality was measured in *szy-13(bs29)* homozygotes, *szy-13(bs29)* heterozygotes, or in *szy-13(bs29)/mat-3(or180)* transheterozygotes at 24°. (C) Immunoblot analysis of embryonic extracts obtained from *zyg-1(it25)* or *zyg-1(it25)*; *szy-13(bs29)* mutants using a SAS-6-specific antibody. α-tubulin (TBA-1/2) was used as a loading control. SAS-6 signal was normalized for loading and relative levels were quantitated. Asterisk denotes a nonspecific band.

Consistent with a role for the APC/C in regulating SAS-6, we identified a *mat-3* loss-of-function allele among our *zyg-1* suppressors. The *mat-3* gene encodes the conserved APC subunit APC8/CDC23. Specifically, we found that the *szy-13(bs29)* mutation, which we had initially mapped to chromosome II (Kemp *et al.* 2007), actually mapped close to *mat-3* on LG III (Figure 6A). Furthermore, the *zyg-1(it25)*; *szy-13(bs29)* strain possessed a missense mutation within the *mat-3* open reading frame; this mutation results in a single amino acid substitution (Arg425Gln) within the conserved TPR repeats of MAT-3. The molecular identity of *mat-3* was confirmed by complementation experiments. First, we showed that the *mat-3(or180)* and *szy-13(bs29)* mutations failed to complement each other for suppression of *zyg-1(it25)* embryonic lethality (Figure 6B). Second, we found that the *mat-3(or180)* and *szy-13(bs29)* mutations also failed to complement each other for suppression of the *zyg-1(it25)* centriole duplication defect. Specifically, we found that both *zyg-1(it25)*; *szy-13(bs29)* and *zyg-1(it25)*; *szy-13 (bs29) +/+mat-3(or180)* strains duplicated centrioles 92% of the time (n = 24 events per strain). We conclude that *bs29* is an allele of *mat-3* and that the loss of APC/C function potently suppresses the *zyg-1(it25)* centriole duplication defect.

Given that MAT-3 is an essential component of the APC/C, and that the APC/C is known to downregulate SAS-6 levels in human tissue culture cells (Strnad *et al.* 2007), we sought to investigate whether MAT-3 might also perform a similar function in worms. Embryonic extracts of *zyg-1(it25)* and *zyg-1(it25)*; *mat-3(bs29)* mutants were analyzed by quantitative immunoblotting using SAS-6-specific antibodies. Contrary to our expectations, we found that the presence of the *mat-3(bs29)* mutation did not result in an increase in the level of SAS-6, suggesting that *C. elegans* embryos, unlike human somatic cells, do not downregulate SAS-6 via the APC/C. Our results further indicate that while EFL-1-DPL-1 regulates centriole duplication by promoting transcription of the core duplication factors, other relevant transcriptional targets exist.

### Embryos lacking EFL-1-DPL-1 activity display cell division defects

Given the role of EFL-1-DPL-1 in modulating the expression of centriole duplication factors, we sought to investigate whether loss of EFL-1-DPL-1-mediated regulation would affect centriole biogenesis or function. Notably, mutation of *dpl-1(bs21)* results in 50–80% embryonic lethality (Kemp *et al.* 2007 and data not shown). To determine the cause of embryonic lethality, we examined early division events in a *dpl-1(bs21)* strain expressing *gfp-spd-2* and *mcherry-his-58* using time-lapse microscopy. Out of 33 embryos, approximately one-third of the embryos exhibited one or more defects during the early embryonic divisions, ranging from centrosome-nucleus attachment defects to delays in the timing of division events. Most intriguingly, we observed the generation of extra centrosomes in two of the embryos (Figure S2). The lack of a stronger effect could either reflect the fact that *dpl-1(bs21)* is a hypomorphic allele or that loss of EFL-1-DPL-1 affects expression of both positive and negative regulators of centriole duplication. Nevertheless, the presence of excess centrosomes in this mutant is consistent with the proposed role for of EFL-1-DPL-1 in limiting expression of SAS-6.

## Discussion

### Transcriptional regulation of centriole duplication factors

Over the past several years, a number of studies have revealed that centriole duplication is regulated in large part by controlling levels of the core centriole assembly factors (Strnad *et al.* 2007; Cunha-Ferreira *et al.* 2009; Rogers *et al.* 2009; Puklowski *et al.* 2011; Peel *et al.* 2012; Čajánek *et al.* 2015). While it is clear that regulated proteolysis plays an important role in achieving the appropriate levels of these proteins, much less is known about how control might be exerted at the level of transcription. Members of the E2F family of transcription factors have been implicated in the control of centriole duplication but the exact role (positive or negative) differs between cell types and E2F family members (Meraldi *et al.* 1999; Saavedra *et al.* 2003; Ma *et al.* 2014). Here, we provide evidence that within a single biological context (the *C. elegans* embryo) the transcriptional regulator complex E2F-DP1 can play both positive and negative roles. Our finding that partial loss-of-function mutations in *dpl-1* and *efl-1* suppress the centriole duplication defect and embryonic lethality of *zyg-1(it25)* introduces the intriguing possibility that one or more centriole duplication factors may be controlled at the level of transcription. Consistent with this, four of the six genes encoding core duplication factors contain putative EFL-1-DPL-1 binding sites in their promoters. Furthermore, these sites have been shown to be occupied by DPL-1 *in vivo* (Kudron *et al.* 2013). To our surprise, however, we found that the mRNA levels of these factors were not increased in either of two *dpl-1* mutants, indicating that they are not negatively regulated by the EFL-1-DPL-1 heterodimer. In fact, mutation of *dpl-1* led to a reproducible decrease in *spd-2*, *zyg-1*, *sas-5*, and *sas-6* RNA levels (Figure 4C). This result is consistent with the findings of (Chi and Reinke 2006) who found that DPL-1 and EFL-1 largely activate transcription within the germ line. Furthermore, our finding that mutation of the DPL-1-bindings sites within the *zyg-1* promoter extinguishes visible expression of a *zyg-1* promoter-driven transgene (Figure 4B) provides additional evidence that EFL-1-DPL-1 promotes expression of centriole duplication genes. This direct positive role for EFL-1-DPL-1 in regulating centriole duplication is therefore counterintuitive when considering that loss of *dpl-1* or *efl-1* suppresses the *zyg-1(it25)* centriole duplication defect. Conceptually, the only way this issue could be reconciled is if EFL-DPL-1 independently functions to negatively regulate centriole assembly, and that partial loss of EFL-1-DPL-1 affects its negative regulatory role more than its positive regulatory role.

### EFL-DPL-1 negatively regulates the level of SAS-6

A key finding of our study is that while loss of EFL-1-DPL-1 activity results in decreased message levels of *sas-6* and other duplication factors, it also results in a significant increase in SAS-6 protein levels. This finding raises two questions: first, how does loss of EFL-1-DPL-1 result in increased expression of SAS-6 and second, how can the elevated level of SAS-6 explain the ability of *dpl-1* or *efl-1* mutations to suppress the *zyg-1(it25*) centriole duplication defect? Since EFL-1-DPL-1 seems to positively regulate transcription of *sas-6*, the increased level of SAS-6 protein in *dpl-1* mutants can only be explained by an indirect mechanism. Given that EFL-1-DPL-1 predominantly activates transcription of genes in the germ line (Chi and Reinke 2006), the most probable mechanism involves EFL-1-DPL-1 promoting the expression of one or more genes that downregulate expression of SAS-6 protein. In such a model, the reduction in EFL-1-DPL-1 activity would affect expression of this negative regulator more than the expression of SAS-6, thus tipping the balance in favor of more SAS-6 protein.

As our results indicate that EFL-1-DPL-1 sets the balance between positive and negative regulators of centriole duplication, one could envision EFL-1-DPL-1 as part of a homeostatic control mechanism that ensures the proper levels of activators and repressors. Such a mechanism would require that the genes encoding the activators and repressors vary in their sensitivity to EFL-1-DPL-1. By adjusting the activity of EFL-1-DPL-1, the cell could vary the relative levels of positive and negative regulators to ensure proper execution of centriole duplication. Experimental manipulation of DPL-1 or EFL-1 levels as reported in the literature could also have the same effect. That is, partial depletion of EFL-1-DPL-1 might result in overduplication, possibly by increasing SAS-6 levels, as seen in our study. In contrast, a strong or complete loss of EFL-1-DPL-1 might lead to a block in duplication as a result of extinguishing transcription of the core centriole duplication genes.

So what is the identity of the negative regulator(s) whose expression depends upon EFL-1-DPL-1 function? To address this question, we looked at the transcriptional targets of EFL-1-DPL-1 as determined by microarray-based expression profiling (Chi and Reinke 2006) and by genome-wide promoter binding profiles (Kudron *et al.* 2013). Among all potential targets in the later study, the APC/C genes *emb-27*, *emb-30*, *gfi-3*/*apc-5*, and *fzy-1* stood out as the most likely candidates. The APC/C is known to negatively regulate SAS-6 protein levels in humans (Strnad *et al.* 2007). Perhaps even more suggestive, we identified a loss-of-function allele of the APC/C gene *mat-3* as a suppressor of *zyg-1(it25)*. However, our analysis indicates that MAT-3 (and the APC/C) functions independently of EFL-1-DPL-1 to regulate centriole biogenesis. Future studies will address whether the APC/C plays a role in controlling the levels of centriole duplication factors in worms as it does in human somatic cells. Along these lines, it is interesting to note that *C. elegans* SAS-6 lacks a KEN box, which is the motif in human SAS-6 recognized by the APC/C coactivator protein Cdh1. Intriguingly, *C. elegans* SAS-5 does have a KEN box and thus the mechanism of APC/C-mediated control of centriole assembly in worms might function through the down regulation of SAS-5 rather than SAS-6. Additional work will be needed to identify the relevant targets of both EFL-1-DPL-1 and the APC/C complex in the centriole duplication pathway.

Finally, how do elevated levels of SAS-6 provide an explanation for the suppression of the *zyg-1(it25)* phenotype? Recently, it has been demonstrated that ZYG-1 recruits SAS-6 to sites of centriole assembly through a direct physical interaction (Lettman *et al.* 2013). The *zyg-1(it25)* mutation might interfere with this recruitment as it results in a nonsynonymous codon change (P442L) within the so-called cryptic polo-box, a domain required to target ZYG-1 to centrioles (Shimanovskaya *et al.* 2014). Thus, the ZYG-1(P442L) protein might be less abundant at centrioles than wild-type ZYG-1, leading to less effective recruitment of SAS-6. Increasing the level of SAS-6 could offset the reduced efficiency of the mutant ZYG-1, leading to sufficient SAS-6 recruitment and successful centriole assembly. While this is the most simplistic interpretation of our results, we have not yet shown that the elevated level of SAS-6 is responsible for suppressing the centriole duplication defect of *zyg-1(it25)*mutants. Thus, it remains possible that suppression arising from loss of EFL-1-DPL-1 activity involves the altered expression of other relevant factors.

Overall, our work indicates that E2F-DP1 plays a complex role in regulating centriole duplication and may serve to establish an equilibrium where the relative levels of positive and negative regulators ensure the faithful duplication of centrioles. Additional work will be needed to uncover the molecular mechanism controlling SAS-6 protein levels as well as the mechanisms by which other EFL-1-DPL-1 targets contribute to the regulation of centriole duplication.

## Supplementary Material

Supporting Information
