# Supplementary material for: The E2F-DP1 Transcription Factor Complex Regulates Centriole Duplication in Caenorhabditis elegans
Source: G3 (Bethesda). 2016 Jan 12;6(3):709–20. doi: 10.1534/g3.115.025577 (PMC4777132; doi:10.1534/g3.115.025577)
Supplement: Supporting Information [file supp_6_3_709__index.html]

The E2F-DP1 Transcription Factor Complex Regulates Centriole Duplication in Caenorhabditis elegans — Supporting Information 

# The E2F-DP1 Transcription Factor Complex Regulates Centriole Duplication in *Caenorhabditis elegans*

## Supporting Information for Goeres-Miller *et al.*, 2016

**Files in this Data Supplement:**

- Figure S1 - Recombination mapping of *dpl-1(bs21)*. (.tif, 880 KB)
- Figure S2 - Early embryogenesis defects in *dpl-1(bs21)* mutants. (.tif, 1149 KB)
- Table S1 - EFL-1-DPL-1 consensus binding sequence and putative EFL-1-DPL-1 binding sites in *zyg-1*, *spd-2*, *sas-5*, and *sas-6* promoters. (.docx, 166 KB)
- Table S2 - Table of strains used in this work. (.docx, 106 KB)
- Table S3 - Oligos used in CRISPR genome editing experiments. (.docx, 66 KB)
- Table S4 - Primers used for qRT-PCR experiments. (.docx, 61 KB)
