# Supplementary figures and images for: The E2F-DP1 Transcription Factor Complex Regulates Centriole Duplication in Caenorhabditis elegans
Source: G3 (Bethesda). 2016 Jan 12;6(3):709–20. doi: 10.1534/g3.115.025577 (PMC4777132; doi:10.1534/g3.115.025577)

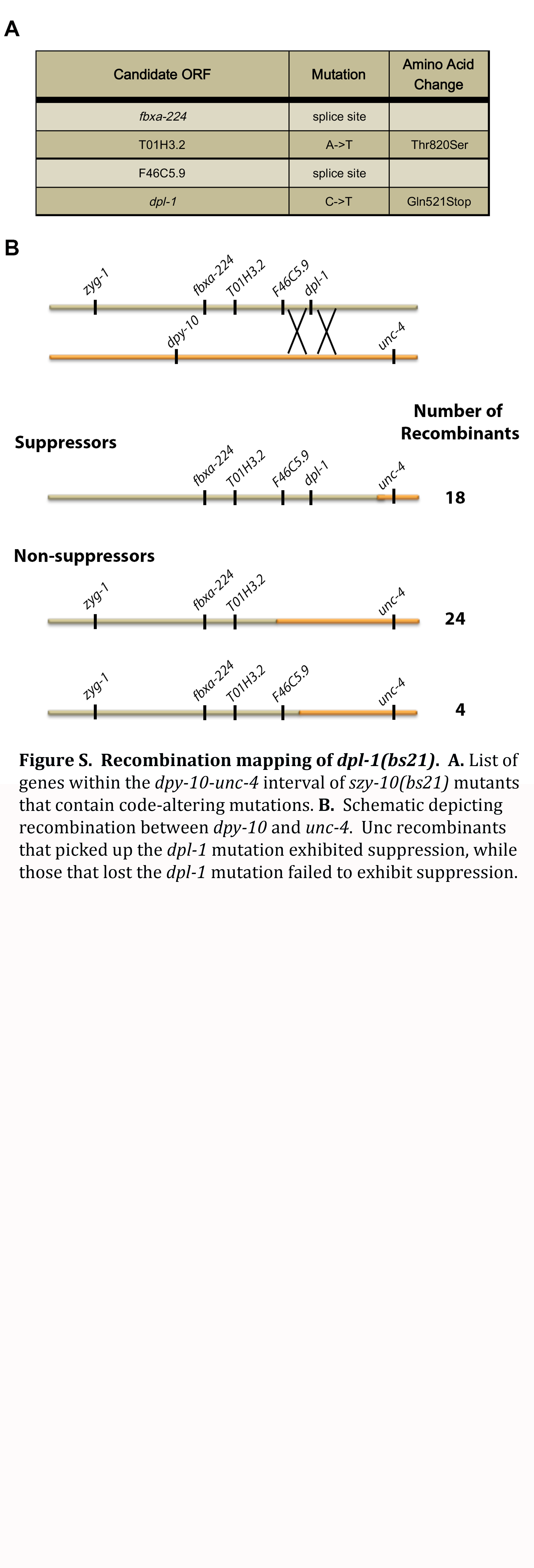

Supplement: Supporting Information [file supp_g3.115.025577_FigureS1.tif]

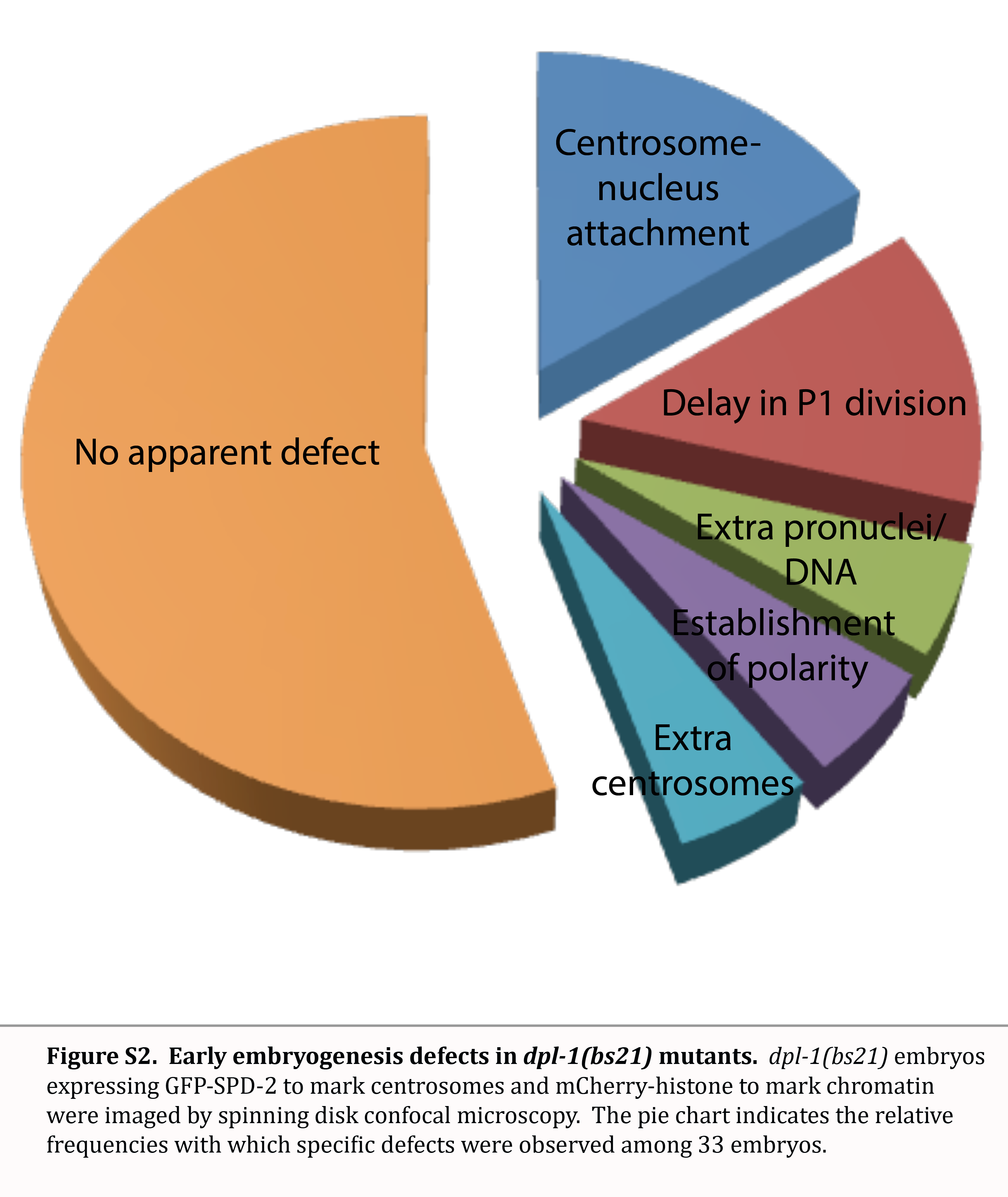

Supplement: Supporting Information [file supp_g3.115.025577_FigureS2.tif]
