# Supplementary material for: The E2F-DP1 Transcription Factor Complex Regulates Centriole Duplication in Caenorhabditis elegans
Source: G3 (Bethesda). 2016 Jan 12;6(3):709–20. doi: 10.1534/g3.115.025577 (PMC4777132; doi:10.1534/g3.115.025577)
Supplement: Supporting Information [file supp_g3.115.025577_TableS1.docx]

**Table S1. EFL-1-DPL-1 consensus binding sequence and putative EFL-1-DPL-1 binding sites in *zyg-1, spd-2, sas-5,* and *sas-6* promoters**

**
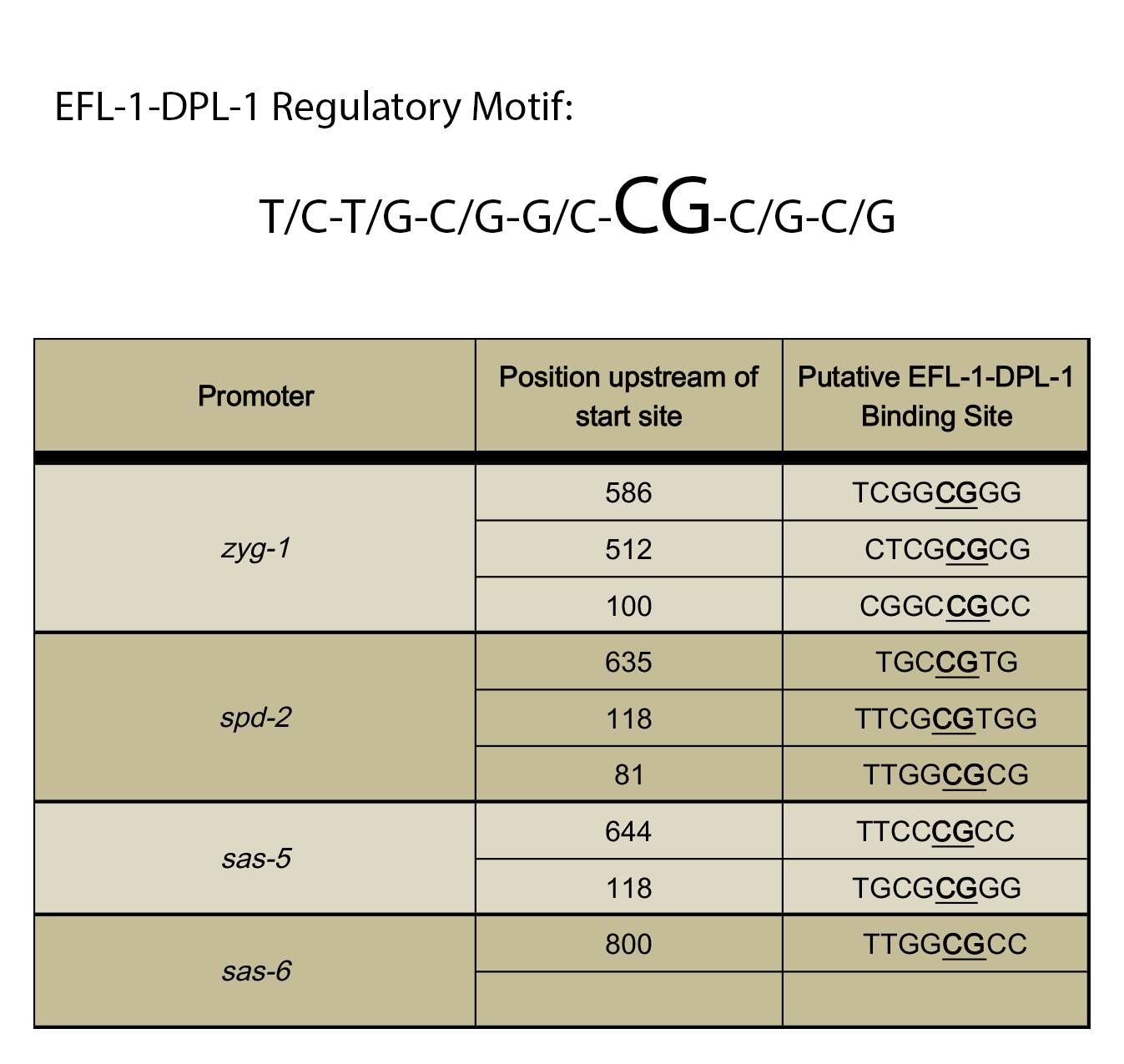
**
