# Supplementary material for: The E2F-DP1 Transcription Factor Complex Regulates Centriole Duplication in Caenorhabditis elegans
Source: G3 (Bethesda). 2016 Jan 12;6(3):709–20. doi: 10.1534/g3.115.025577 (PMC4777132; doi:10.1534/g3.115.025577)
Supplement: Supporting Information [file supp_g3.115.025577_TableS2.docx]

| **Table S2. *C. elegans* strains used in this study** | |
| --- | --- |
| Strain Name | Genotype |
| N2 | wild type |
| OC14 | *zyg-1(it25) II* |
| OC489 | *zyg-1(it25) dpl-1(bs21) II* |
| OC248 | *zyg-1(it25); efl-1(bs22) V* |
| OC495 | *zyg-1(it25) II; bsIs2 [pCK6.1: unc-119(+) ppie-1-gfp-spd-2]; ltIs37 [pAA64: unc-119(+) ppie-1-mcherry-his-58]* |
| OC492 | *zyg-1(it25) szy-10(bs21) II; bsIs2 [pCK6.1: unc-119(+) ppie-1-gfp-spd-2]; ltIs37 [pAA64: unc-119(+) ppie-1-mcherry-his-58]* |
| OC614 | *zyg-1(it25) dpy-10(e128) II* |
| OC602 | *zyg-1(it25) dpy-10(e128) unc-4(e120) II* |
| OC616 | *zyg-1(it25) dpy-10(e128) dpl-1(bs21) II* |
| OC615 | *zyg-1(it25) dpy-10(e128) F46C5.9(bs54, bs55) dpl-1(bs21) II* |
| OC580 | *zyg-1(it25) unc-4 (e120) II* |
| OC584 | *zyg-1(it25) F46C5.9(bs54, bs55) dpl-1(bs21) unc-4(e120) II* |
| OC583 | *zyg-1(it25) F46C5.9(bs54, bs55) unc-4(e120) II* |
| YL390 | *unc-119(ed3)III; vrIs48 [ppie-1-dpl-1-gfp-flag-dpl-1 3'utr; unc-119(+)]* |
| JJ1549 | *efl-1(se1) V* |
|  | *zyg-1(it25); efl-1(se1) V* |
| OC248 | *zyg-1(it25ts)II; efl-1(bs22) V* |
| OC617 | *efl-1(bs22) V* |
| OC790 | *zyg-1(it25) II; efl-1(bs22, bs102) V* |
| OC791 | *zyg-1 (it25) II; efl-1 (bs103) V* |
| OC491 | *dpl-1(bs21)* |
| MT11147 | *dpl-1(n3643) II* |
| OC723 ^a^ | *bsSi9 [pKO113: unc-119(+) pzyg-1(wt)-gfp-his-58-zyg-1 3’utr]I; unc-119(ed3)III* |
| OC725 ^a^ | *bsSi11 [pKO114: unc-119(+) pzyg-1(3X mut)-gfp-his-58-zyg-1 3’utr]I; unc-119(ed3)III* |
| OC184 | *zyg-1(it25ts)II; mat-3(bs29) III* |
| OC627 | *zyg-1(it25ts)II; mat-3(or180) III* |
| OC644 | *zyg-1(it25);mat-3(bs29); bsIs2 [pCK5.5: pie-1-gfp-spd-2];*  *ltIs37[pAA64: unc-119(+) pie-1-mcherry-his58]* |
| OC500 | *szy-10(bs21) II; bsIs2 [pCK6.1: unc-119(+) pie-1-gfp-spd-2]; ltIs37 [pAA64: unc-119(+) pie-1-mcherry-his-58]* |

^a^ OC723 carries a transgene with a wild-type *zyg-1* promoter and OC725 carries the same transgene integrated in the same position within the genome but with a zyg-1 promoter mutated for the three EFL-1-DPL-1 binding sites.
