# Supplementary material for: The E2F-DP1 Transcription Factor Complex Regulates Centriole Duplication in Caenorhabditis elegans
Source: G3 (Bethesda). 2016 Jan 12;6(3):709–20. doi: 10.1534/g3.115.025577 (PMC4777132; doi:10.1534/g3.115.025577)
Supplement: Supporting Information [file supp_g3.115.025577_TableS3.docx]

| **Table S3: Oligos used in CRISPR genome editing** | |
| --- | --- |
| Oligo Name Oligo Sequence | |
| *efl-1* gRNA1^a^ | 5’-GTGTTACCTGAAAGATCCCTCgttttagagctagaaatagcaagt-3’ |
| *efl-1* gRNA2 ^a^ | 5’-GAAACGATGGCGGCTCGGAGgttttagagctagaaatagcaagt-3’ |
|  |  |
| *efl-1* repair 1^b^ | 5’-aaaaaattgcagCAAGTCGAGATACACGGTGGACCATCTTGGTATT ACCTGAAAGATCCCTCTGGACCCCTCCGAGCCGCCATCGTTTCC AACCATGAGC-3’ |
| *efl-1* repair 2^c^ | 5’-aaaaaattgcagCAAGTCGAGATACACGGTGGACCATCTTGGTGTT ACCTGAAAGATCCCTCTGGACCCCTCCGAGCCGCCATCGTTTCC AACCATGAGC-3’ |
| *dpy-10 gRNA* | 5’-GCTACCATAGGCACCACGAG-3’ |
| *dpy-10 (cn64)* repair template | 5’-CACTTGAACTTCAATACGGCAAGATGAGAATGACTGGAAACC GTACCGCATGCGGTGCCTATGGTAGCGGAGCTTCACATGGCTTCA  GACCAACAGCCTAT-3’ |

^a^ Lower case sequence added for addition to pDD162 with Q5 kit

^b^ Creates *efl-1(bs22)*

^c^ Creates *efl-1(wt)*
