# Supplementary material for: The E2F-DP1 Transcription Factor Complex Regulates Centriole Duplication in Caenorhabditis elegans
Source: G3 (Bethesda). 2016 Jan 12;6(3):709–20. doi: 10.1534/g3.115.025577 (PMC4777132; doi:10.1534/g3.115.025577)
Supplement: Supporting Information [file supp_g3.115.025577_TableS4.docx]

| **Table S4: Primers used in qRT-PCR experiments** | |
| --- | --- |
| Primer Name | Primer Sequence |
| *tba-1_225_FP* | 5’-CTGTTGTTGATGAGATCCGCACTGG- 3’ |
| *tba-1_438_RP* | 5’-TCAGCGAGGCGACGGATTC - 3’ |
|  |  |
| *dpl-1_61_FP* | 5’ –CTACGATCCCCGTATCGGCC- 3’ |
| *dpl-1_193_RP* | 5’ –GCCCACAGGTTCATTGTGACTCTC- 3’ |
| *zyg-1_397_FP* | 5’-GTGCGAAGGAGGATCTCTTCAAGC- 3’ |
| *zyg-1_547_RP* | 5’-CACATTACCAGCCGAGAGGTCTC- 3’ |
|  |  |
| *sas-5_1091_FP* | 5’- GCTGAATGTGATGCTAACAGAGCTGC – 3’ |
| *sas-5_1542_*RP | 5’- GTACGGTATGAGTCAGGAGCACG – 3’ |
|  |  |
| *sas-6_690_FP* | 5’-GCAAGCCTACGGCAGAAATGTG - 3’ |
| *sas-6_823_RP* | 5’-CTTTCCTCTTTCACCAGCTCCACC- 3’ |
|  |  |
| *spd-2_1070_FP* | 5’- CTGTTCGCAGCATTAGAAGAAGCTCG – 3’ |
| *spd-2_1381_RP* | 5’- GCAGTTGTGTTGTCGTTGGACG – 3’ |
